# Low Dose Naltrexone in Depression: A Mechanistic Systematic Review of Convergent Neuroimmune Suppression and Endogenous Opioid Plasticity as a Mechanistic Framework

**DOI:** 10.1192/bjo.2026.11142

**Published:** 2026-06-30

**Authors:** Gaurav Uppal, Faris Basheer Mohamed, Naima Gul, Betsy Babu, Kasthuri Pandian

**Affiliations:** 1 Satyam Hospital, Ludhiana,India; 2 Disha Deaddiction and Neuropsychiatric clinic, Jammu, India; 3 Leeds Community Healthcare Trust, Leeds, United Kingdom; 4 North London Forensic Service, Chase Farm Hospital, Enfield, London, United Kingdom; 5 MVJ Medical College Research Hospital, Bangalore, India

## Abstract

**Aims:**

There is increasing evidence that suggests that different subtypes of major depressive disorders, particularly treatment-resistant depression and anhedonia-predominant forms, have dysregulation in reward processing and chronic neuroimmune activation. Low-dose naltrexone (LDN), which is administered at low doses (1–5 mg/day), modifies both the signalling from the body’s endogenous system and the inflammatory pathways of microglia and thus can be used as a potential therapeutic for depression. This mechanistic systematic review aims to evaluate the dual mechanism of Low Dose Naltrexone in Treatment-Resistant Depression. This study aims to provide an analysis of the human clinical and biological effects of low dose naltrexone (LDN) in depression based upon a mechanistic convergence framework.

**Methods:**

Human studies examining the effects of LDN on outcomes such as mood, fatigue, pain, quality of life, inflammatory markers, or opioid-related variables, published in PubMed, EMBASE, Scopus, Web of Science, the Cochrane Library, or Google Scholar, wereidentified. The quality of randomized controlled trials was evaluated according to the Risk of Bias 2 tool, and the quality of non-randomized studies was evaluated according to the Risk Of Bias In Non-randomized Studies–Instrument (ROBINS-I). Results from studies of human subjects were synthesized narratively using a mechanism-based approach.

**Results:**

Improvements in quality of life, fatigue, and mood-related symptoms in disorders characterized by immune activation and central sensitization (e.g. fibromyalgia, multiple sclerosis) were observed across randomized trials, observational cohorts, and case reports after administration of LDN. Biomarkers of inflammation (pro-inflammatory cytokine levels) were lower in human studies after administration of LDN. Indirect indicators of enhancement of endogenous opioid function (e.g. improved pain tolerance, decreased consumption of analgesics and psychotropics) were also reported in human studies after administration of LDN. The mechanisms by which LDN exerts these beneficial effects occur in the same patient population and therefore suggest a biological convergence of immune suppression and opioid-mediated reward modulation.

**Conclusion:**

The results from the literature support a “Dual Mechanistic Model” where LDN suppresses inflammation by activated microglia and at the same time increases signalling of endogenous opioids, both of which have been implicated in the pathophysiology of depression and anhedonia. Thus there is a strong mechanistic basis for testing LDN as a treatment for patients who have failed to respond to current treatments for depression.

